# Supplementary material for: Sensory nerve niche regulates mesenchymal stem cell homeostasis via FGF/mTOR/autophagy axis
Source: Nat Commun. 2023 Jan 20;14:344. doi: 10.1038/s41467-023-35977-4 (PMC9859800; doi:10.1038/s41467-023-35977-4)
Supplement: Supplementary file 1 — Supplementary Information [file 41467_2023_35977_MOESM1_ESM.pdf]

Sensory nerve niche regulates mesenchymal stem cell homeostasis via FGF/mTOR/autophagy axis

This document includes:

Supplementary Fig.1 to Supplementary Fig. 8

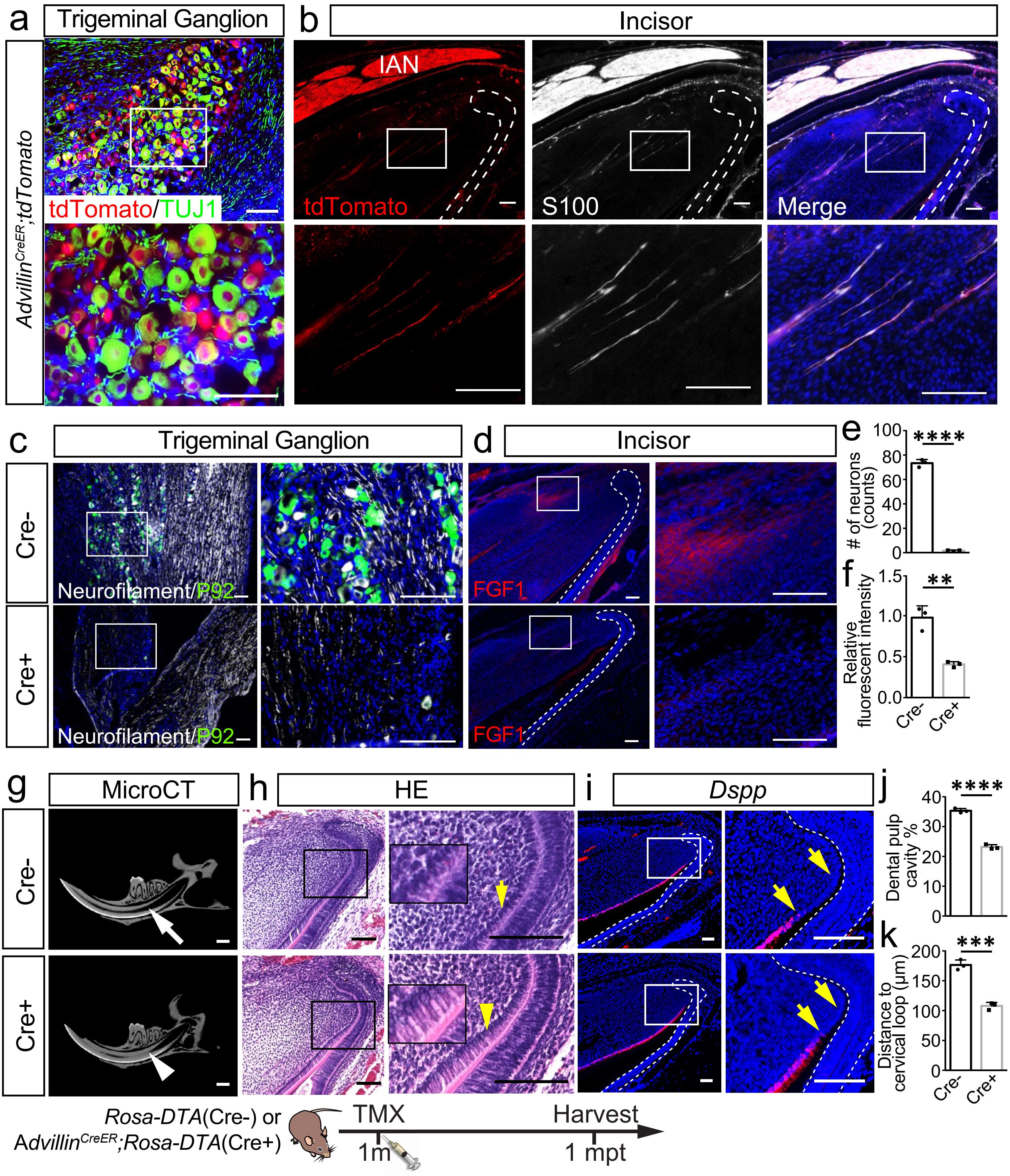

a
Trigeminal Ganglion
Advillin^{CreER};tdTomato
tdTomato/TUJ1
b
Incisor
IAN
tdTomato
S100
Merge
c
Trigeminal Ganglion
Cre-
Cre+
Neurofilament/P92
d
Incisor
FGF1
e
# of neurons (counts)
****
f
Relative fluorescent intensity
**
Cre-
Cre+
g
MicroCT
h
HE
i
Dspp
j
Dental pulp cavity %
****
k
Distance to cervical loop (μm)
***
Rosa-DTA(Cre-) or
Advillin^{CreER};Rosa-DTA(Cre+)
TMX
Harvest
1m
1 mpt

Supplementary Fig. 1: Deletion of sensory nerve leads to abnormal dentin formation.

a Colocalization of tdTomato and nerve marker TUJ1 in the trigeminal ganglion of *Advillin*^{CreER}*;tdTomato* mice 7 days after induction. **b** *Advillin* targeted inferior alveolar nerve (IAN) and nerve fibers in the incisor of *Advillin*^{CreER}*;tdTomato* mice. Expression of tdTomato in IAN and nerves in the incisor, and colocalized with nerve marker S100. **c** Sensory neurons stained with P92 in the trigeminal ganglion of control and *Advillin*^{CreER}*;Rosa-DTA* mice one month after tamoxifen induction. **d** Expression of FGF1 decreased in the incisor one month after sensory nerve deletion. **e** Quantification of neuron numbers in trigeminal ganglion in control and *Advillin*^{CreER}*;Rosa-DTA* mice. P<0.0001. **f** Quantification of relative fluorescent intensity of FGF1 in control and mutant mice. P=0.0026. **g** CT scanning of control and *Advillin*^{CreER}*;Rosa-DTA* mice one month after tamoxifen induction. White arrow points to dental pulp cavity; white arrowhead points to narrowed pulp cavity. **h** Histological analysis of control and *Advillin*^{CreER}*;Rosa-DTA* mice one month after induction. Yellow arrow points to pre-odontoblast; yellow arrowhead points to abnormal pre-odontoblast. **i** *Dspp* expression in control and mutant mice. Yellow arrow points to the distance between the bending point of the cervical loop and the initiation of odontoblast differentiation. **j** Quantification of dental pulp cavity percentage in control and mutant mice. P<0.0001. **k** Quantification of distance of *Dspp*$^{+}$ cells to cervical loop in control and mutant mice. P=0.0003. For **e, f, j** and **k** n=3, each data point represents one animal, with unpaired Student's t-test performed. All data are expressed as the mean±SD. Source data are provided as a Source Data file. **P<0.01, ****P<0.0001. Each experiment was repeated independently three times. White dotted line outlines cervical loop. Cre-: *Rosa-DTA*; Cre+: *Advillin*^{CreER}*;Rosa-DTA.* Scale bars, **g** 1mm; others, 100μm.

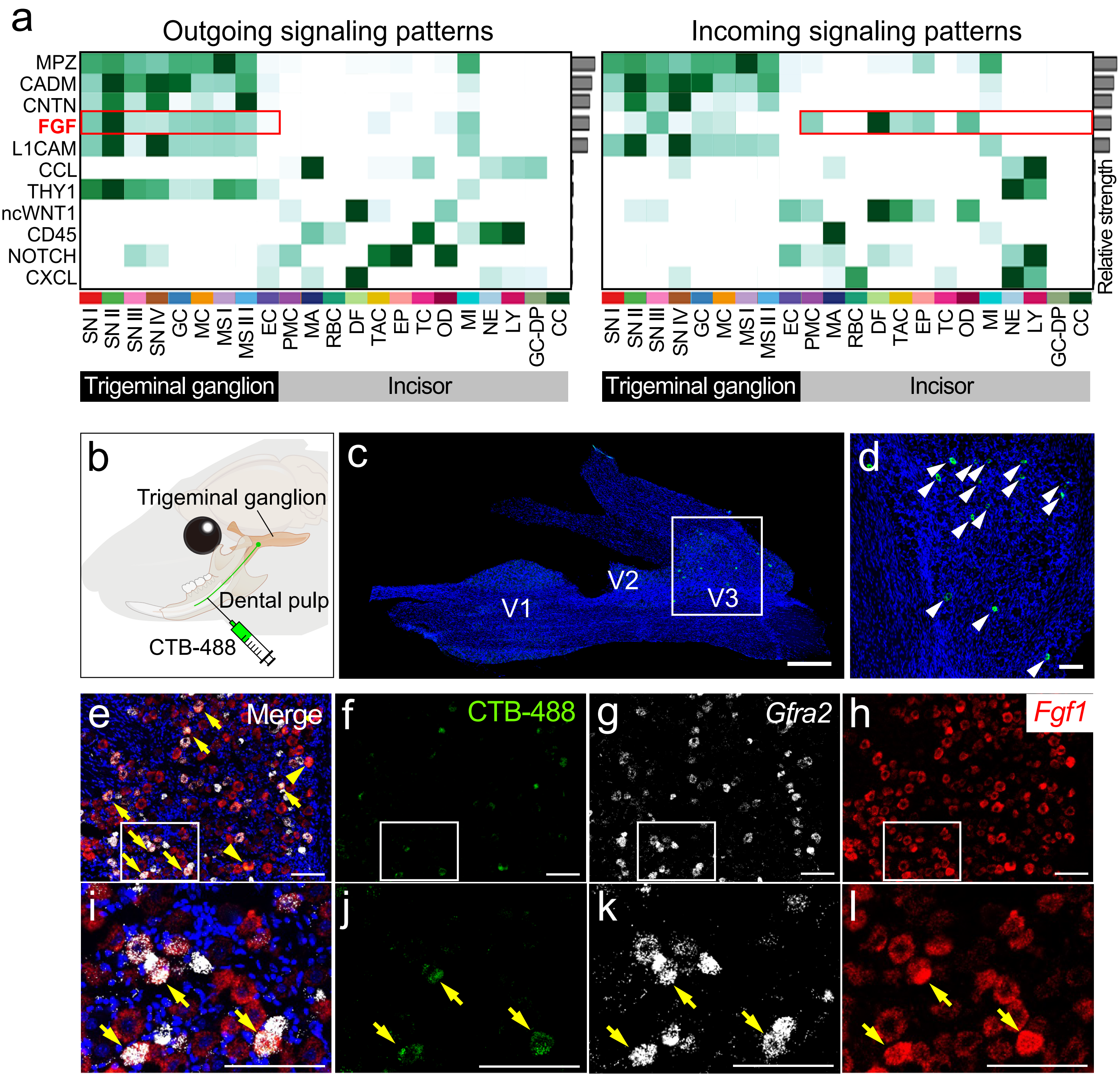
a
Outgoing signaling patterns
Incoming signaling patterns
MPZ
CADM
CNTN
FGF
L1CAM
CCL
THY1
ncWNT1
CD45
NOTCH
CXCL
SN I
SN II
SN III
SN IV
GC
MC
MS I
MS III
EC
PMC
MA
RBC
DF
TAC
EP
TC
OD
MI
NE
LY
GC-DP
CC
Trigeminal ganglion
Incisor
Relative strength
0
1
b
Trigeminal ganglion
Dental pulp
CTB-488
c
V1
V2
V3
d
e
Merge
f
CTB-488
g
Gfra2
h
Fgf1
i
j
k
l

Supplementary Fig. 2: FGF1 is an important ligand secreted from sensory neurons.

a Significant signaling pathways involved in the interaction between the trigeminal ganglion and incisor of an adult mouse. SNI-IV, sensory neuron type I-IV; GC, glia cell; PMC, proximal mesenchymal cell (MSC region); MC, meningeal cell; MS, myelinating Schwann cell; nMS, non-myelinating Schwann cell; MA, macrophage; RBC, red blood cell; DF, dental follicle cell; TAC, TA cell; TC, T cell; OD, odontoblast; MI, microglia; NE, neutrophil; LY, lymphocyte; EC, endothelial cell; GC-DP, glia cell in dental pulp; CC, cycling cell. **b** Schematic illustrating the retrograde fluorescent tracing. **c-d** Fluorescent cells in the trigeminal ganglion. CTB-488$^+$ neurons were exclusively detected in the V3 branch of the trigeminal nerve. V1, V2 and V3 indicate the three branches of the trigeminal nerve. White arrowheads point to CTB-488$^+$ neurons. **e-l** Mechanosensation neurons innervating incisor secrete FGF1. **e and i** Colocalization of CTB-488, *Gfra2* and *Fgf1* in V3 of trigeminal ganglion. **f and j** CTB-488$^+$ neurons in trigeminal ganglion. **g and k** Expression of *Gfra2* in trigeminal ganglion. **h and l** Expression of *Fgf1* in trigeminal ganglion. Yellow arrows indicate CTB-488$^+$/*Gfra2*$^+$/*Fgf1*$^+$ neurons; yellow arrowheads indicate CTB-488$^+$/*Fgf1*$^+$ neurons. Scale bars, **c**, 500μm; others, 100μm.

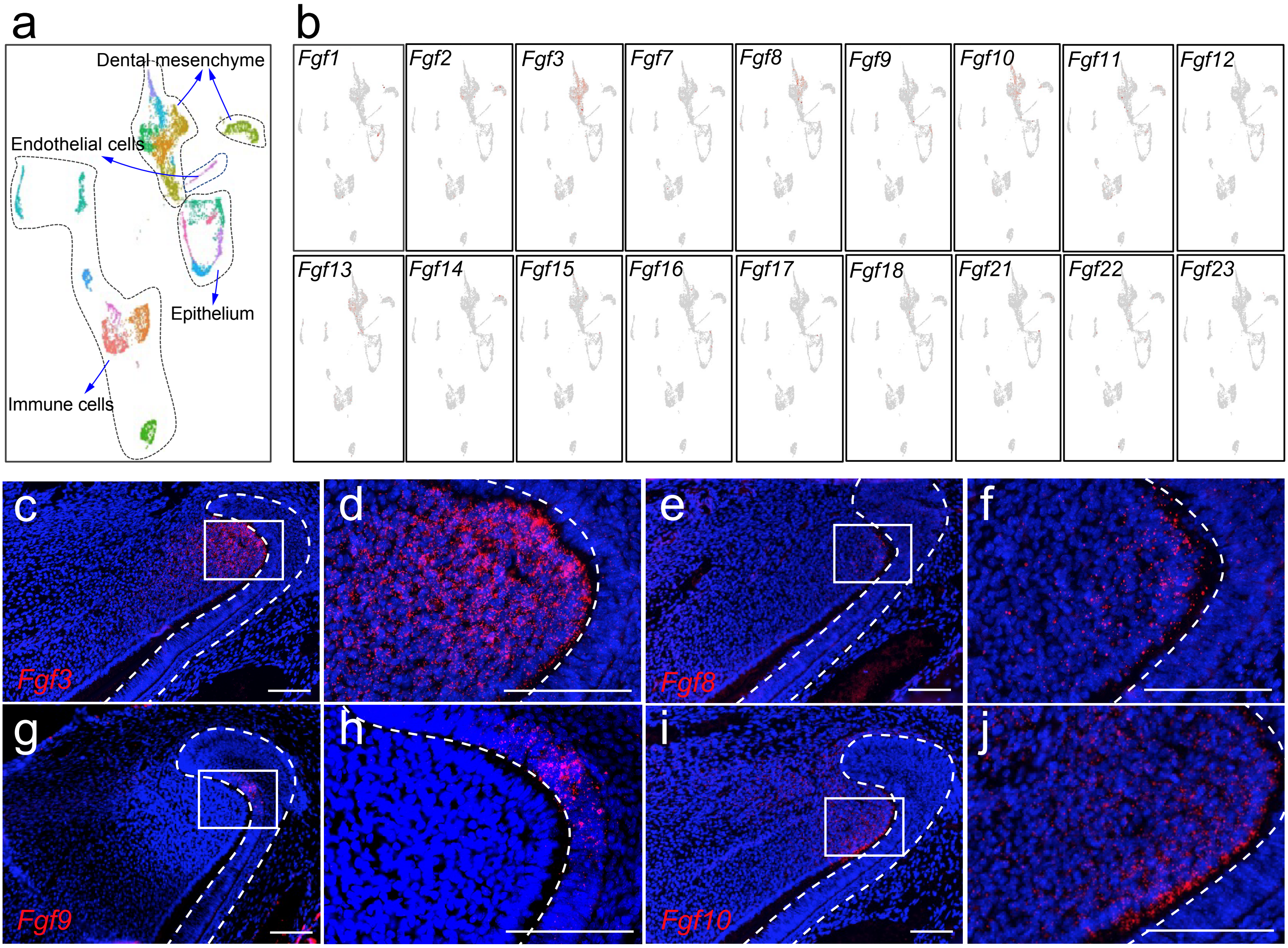
a
Dental mesenchyme
Endothelial cells
Epithelium
Immune cells
b
Fgf1
Fgf2
Fgf3
Fgf7
Fgf8
Fgf9
Fgf10
Fgf11
Fgf12
Fgf13
Fgf14
Fgf15
Fgf16
Fgf17
Fgf18
Fgf21
Fgf22
Fgf23
c
Fgf3
d
e
Fgf8
f
g
Fgf9
h
i
Fgf10
j

Supplementary Fig. 3: FGF ligands in adult mouse incisor.

a-b Feature plot of FGF ligands in adult mouse incisor. **c-d** RNAscope staining of *Fgf3* in the incisor. **e-f** RNAscope staining of *Fgf8* in the incisor. **g-h** Expression of *Fgf9* in the incisor. **i-j** Expression of *Fgf10* in the incisor. White dotted line outlines cervical loop. Scale bars, 100μm.

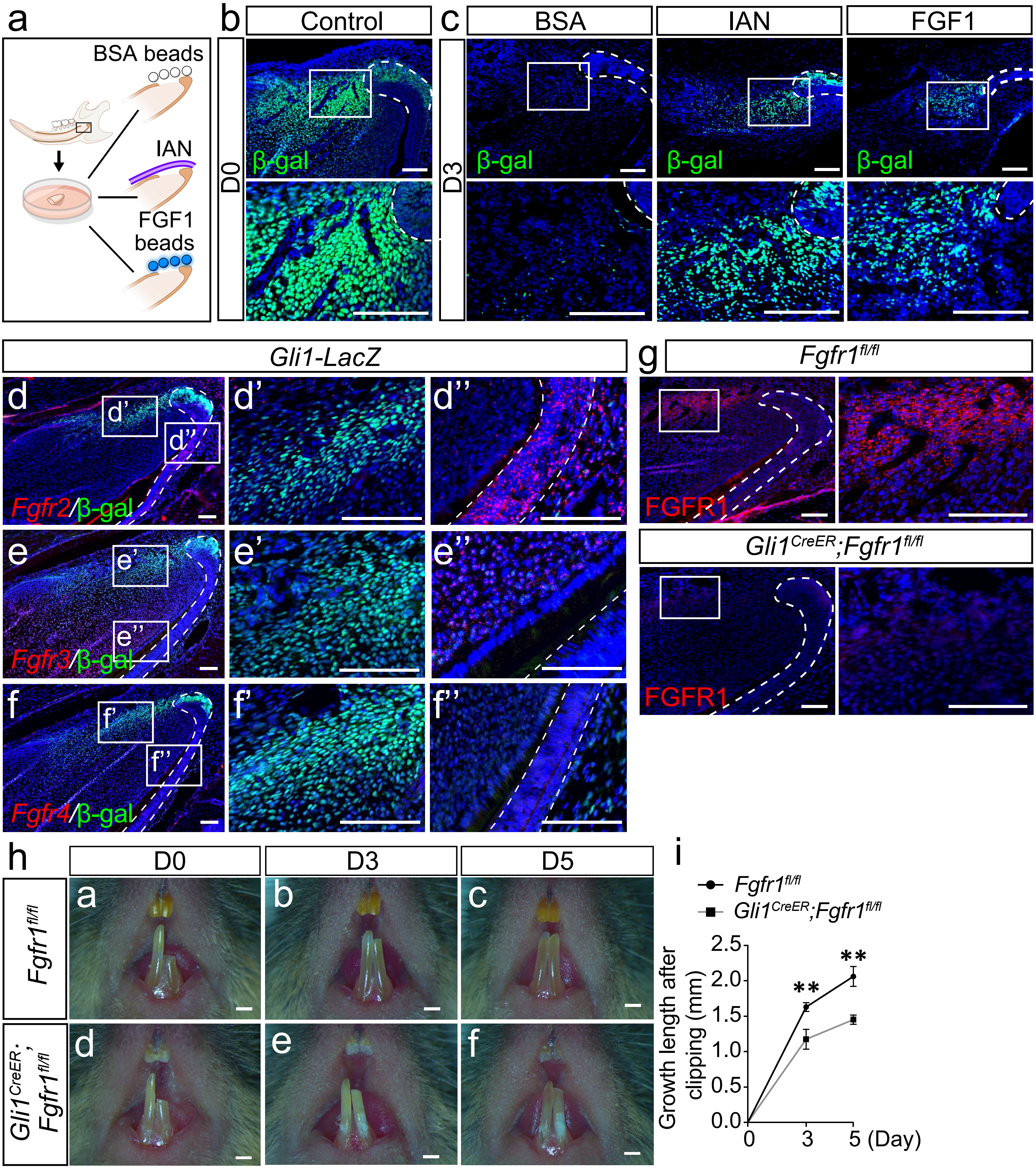

a
BSA beads
IAN
FGF1 beads
b
Control
D0
β-gal
c
BSA
IAN
FGF1
D3
β-gal
β-gal
β-gal
Gli1-LacZ
d
d'
d''
Fgfr2/β-gal
e
e'
e''
Fgfr3/β-gal
f
f'
f''
Fgfr4/β-gal
g
Fgfr1 fl/fl
FGFR1
Gli1 CreER;Fgfr1 fl/fl
FGFR1
h
D0
D3
D5
Fgfr1 fl/fl
Gli1 CreER;Fgfr1 fl/fl
a
b
c
d
e
f
i
Fgfr1 fl/fl
Gli1 CreER;Fgfr1 fl/fl
Growth length after clipping (mm)
2.5
2.0
1.5
1.0
0.5
0.0
0
3
5 (Day)
**
**

Supplementary Fig. 4: FGF1 secreted from sensory nerves directly acts on MSCs via FGFR1.

a-c FGF1 is crucial for MSC maintenance. **a** Schematic of the incisor explant culture. The proximal ends of *Gli1-LacZ* mouse incisors were cultured with BSA beads, IAN and recombinant FGF1 beads respectively. **b** Expression of *Gli1*$^+$ cells in the proximal end of the incisor. **c** *Gli1*$^+$ cells labeled with β-gal in incisor explant with BSA beads, IAN surrounding the proximal end or recombinant FGF1 beads cultured for 3 days respectively.

d-f Expression of FGFRs in the incisor. **d** Expression patterns of *Fgfr2* and β-gal in the incisors of *Gli1-LacZ* mice. **e** *Fgfr3* is expressed in the sub-odontoblast layer and in partial pulp cells, but is barely detectable in the proximal end of the incisor. **f** *Fgfr4* expression is undetectable in the incisor.

g FGFR1 was deleted in $Gli1^{CreER};Fgfr1^{fl/fl}$ mice. The expression of FGFR1 is shown in control and $Gli1^{CreER};Fgfr1^{fl/fl}$ mice.

h Tissue repair is impaired after FGF signaling is deleted in MSCs. The incisor was clipped and the subsequent injury repair was observed in control and $Gli1^{CreER};Fgfr1^{fl/fl}$ mice on D3 and D5 after clipping. The amount of incisor growth in control and $Gli1^{CreER};Fgfr1^{fl/fl}$ mice was analyzed on D3 and D5 after clipping. D3: P=0.0069; D5: P=0.0024. N=3, each data point represents one animal, with unpaired Student's t-test performed. All data are expressed as the mean±SD. Source data are provided as a Source Data file. **P<0.01, ****P<0.0001. Each experiment was repeated independently three times. White dotted line shows the cervical loop. Scale bars, **h** 1mm; others, 100μm.

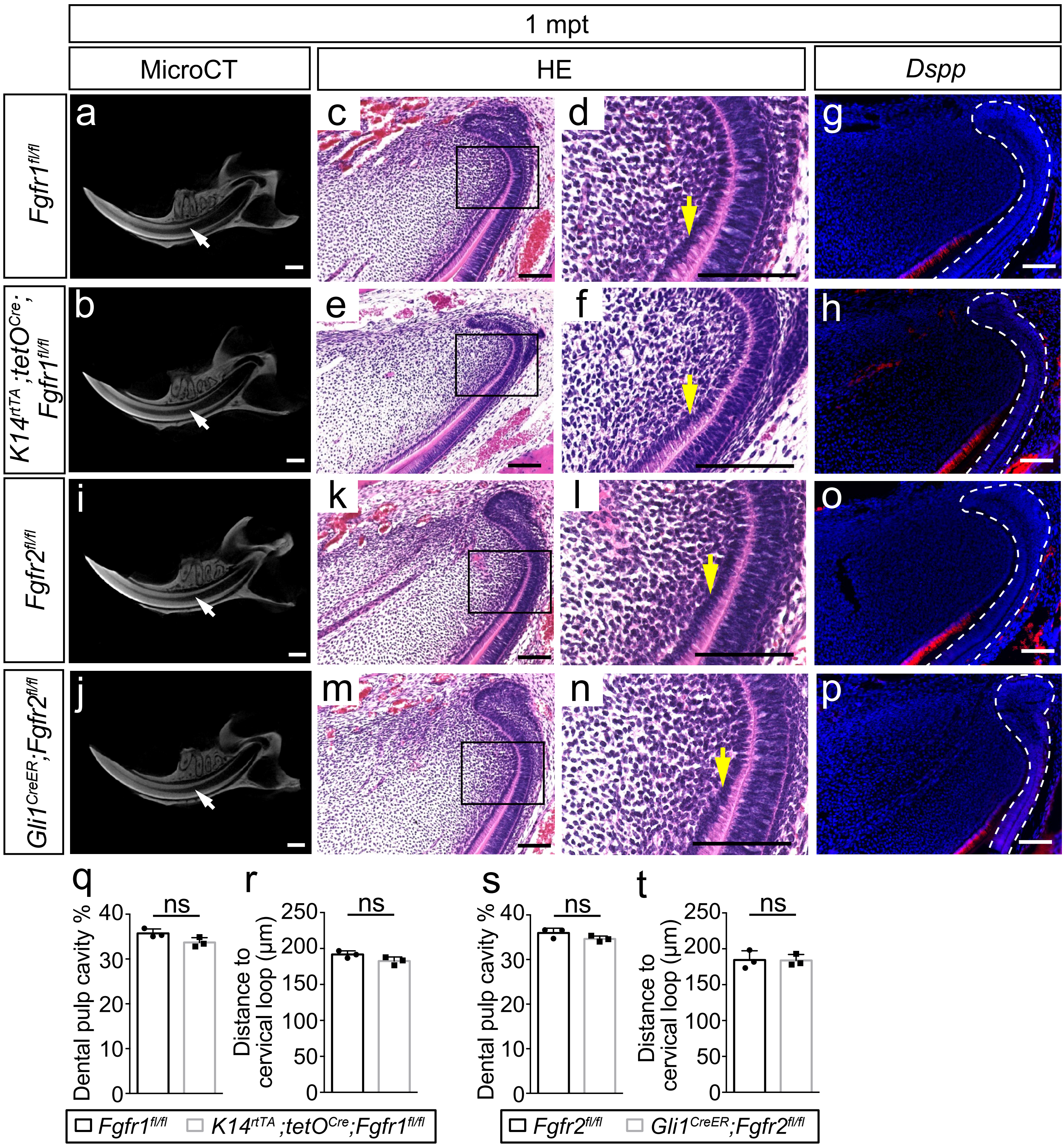

1 mpt
MicroCT
HE
Dspp
Fgfr1fl/fl
K14rtTA;tetOCre;Fgfr1fl/fl
Fgfr2fl/fl
Gli1CreER;Fgfr2fl/fl
a
b
c
d
e
f
g
h
i
j
k
l
m
n
o
p
q
r
s
t
Dental pulp cavity %
Distance to cervical loop (µm)
ns
0
10
20
30
40
50
100
150
200
250
Fgfr1fl/fl
K14rtTA;tetOCre;Fgfr1fl/fl
Fgfr2fl/fl
Gli1CreER;Fgfr2fl/fl

Supplementary Fig. 5: Neither depletion of FGFR1 in epithelium nor depletion of FGFR2 in MSCs affect mesenchymal homeostasis.

a-h No obvious change in dentin formation is observed when FGFR1 is depleted from the epithelium. **a-b** CT images of control and $K14^{rtTA}$;$tetO^{Cre}$;$Fgfr1^{fl/fl}$ mice one month after induction. The morphology with histological analysis (**c-f**) and differentiation of odontoblasts (**g-h**) showed no obvious differences between groups. **q** Quantification of dental pulp cavity percentage in control and mutant mice. P=0.0712. **r** Quantification of distance of $Dspp^{+}$ cells to cervical loop in control and mutant mice. P=0.1044.

i-p No obvious change in dentin formation is observed when FGFR2 is depleted in MSCs. **i-j** CT images of control and $Gli1^{CreER}$;$Fgfr2^{fl/fl}$ mice one month after induction. The odontoblast morphology (**k-n**) and differentiation (**o-p**) are unaffected in $Gli1^{CreER}$;$Fgfr2^{fl/fl}$ mice. **s** Quantification of dental pulp cavity percentage in control and mutant mice. P=0.1428. **t** Quantification of distance of $Dspp^{+}$ cells to cervical loop in control and mutant mice. P=0.9412. White arrow points to dental pulp cavity; yellow arrow points to pre-odontoblast. For **q-t** n=3 and each data point represents one animal, with unpaired Student's t-test performed. All data are expressed as the mean±SD. Source data are provided as a Source Data file. **P<0.01, ****P<0.0001. Each experiment was repeated independently three times. White dotted line outlines cervical loop. Scale bars, **a-b** and **i-j** 1mm; others, 100μm.

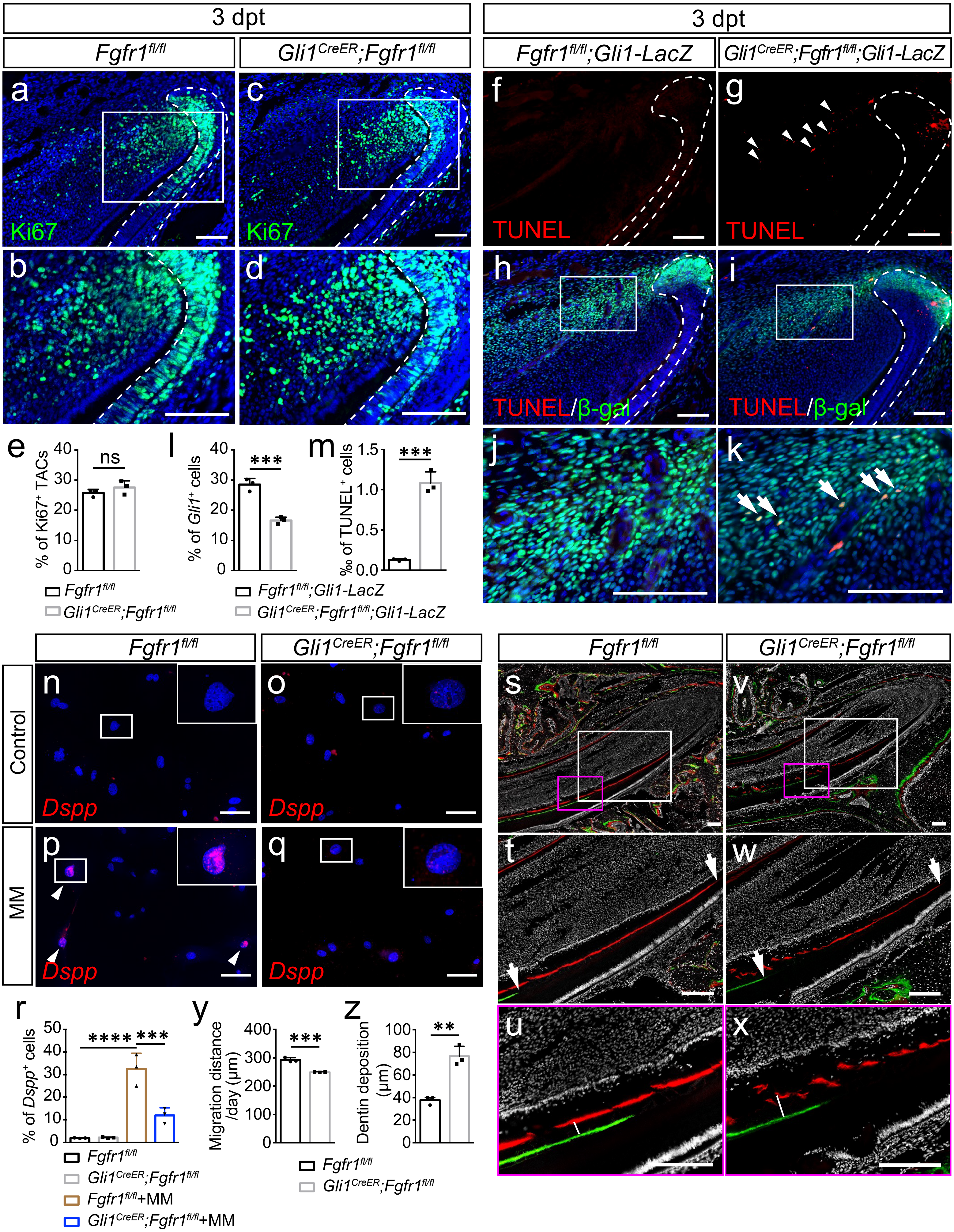

3 dpt
Fgfr1fl/fl
Gli1CreER;Fgfr1fl/fl
Fgfr1fl/fl;Gli1-LacZ
Gli1CreER;Fgfr1fl/fl;Gli1-LacZ
Ki67
TUNEL
TUNEL/β-gal
% of Ki67+ TACs
ns
% of Gli1+ cells
‰ of TUNEL+ cells
Control
MM
Dspp
% of Dspp+ cells
Migration distance /day (μm)
Dentin deposition (μm)
Fgfr1fl/fl+MM
Gli1CreER;Fgfr1fl/fl+MM

Supplementary Fig. 6: Loss of FGF signaling in MSCs disturbs mesenchymal tissue homeostasis.

a-k Apoptosis leads to decreased MSCs. **a-d** TACs stained with Ki67 in control and *Gli1^{CreER};Fgfr1$^{fl/fl}$* mice 3 days after induction. **e** Quantification of Ki67^{+} TACs in control and mutant mice. P=0.2817. **f-m** Increased apoptosis in *Gli1^{+}* cells in *Gli1^{CreER};Fgfr1$^{fl/fl}$;Gli1-LacZ* mice. **f-g** Apoptosis detected with TUNEL staining in control and *Gli1^{CreER};Fgfr1$^{fl/fl}$*;*Gli1-LacZ* mice. White arrowheads point to TUNEL^{+} cells. **h-k** TUNEL and β-gal staining in control and mutant mice. White arrows point to co-localization of TUNEL^{+} cells and *Gli1^{+}* cells. **l** Quantification of *Gli1^{+}* cells in the mesenchyme. P=0.0009. **m** Quantification of TUNEL^{+} cells in the mesenchyme. P=0.0003. N=3, each data point represents one animal, with unpaired Student's t-test performed.

n-r Decreased odontoblastic differentiation of MSCs with FGF signaling depletion. Expression of *Dspp* in cells from control and *Gli1^{CreER};Fgfr1$^{fl/fl}$* mice, after culturing for 3 days with medium (**n-o**) or mineralized medium (MM) (**p-q**). White arrowhead points to *Dspp^{+}* cells. **r** Quantification of Ki67^{+} TACs in control and mutant mice. *Fgfr1$^{fl/fl}$* versus *Gli1^{CreER};Fgfr1$^{fl/fl}$*: P<0.0001; *Fgfr1$^{fl/fl}$*+MM versus *Gli1^{CreER};Fgfr1$^{fl/fl}$*+MM: P=0.0009. N=3 each data point represents one biological replicate, with one-way ANOVA analysis.

s-z Decreased migration of odontoblasts with FGF signaling depletion. **s and v** With double labeling by calcein and Alizarin red injection, we can observe the dynamics of dentin formation. **t and w** The length between red and green fluorescence indicates migration of odontoblasts within 5 days. **u and x** The thickness between red and green fluorescence shows the deposition of dentin. White arrows point to the length of odontoblast migration; white lines indicate the thickness of dentin. **y** Quantification of migration distance per day. P=0.0007. **z and e** Quantification of dentin deposition. P=0.0022. N=3, each data point represents one animal, with unpaired Student's t-test performed. All data are expressed as the mean±SD. Source data are provided as a Source Data file. **P<0.01, ****P<0.0001. Each experiment was repeated independently three times. White dotted line shows the cervical loop. Scale bars, **s and v** 200μm; others, 100μm.

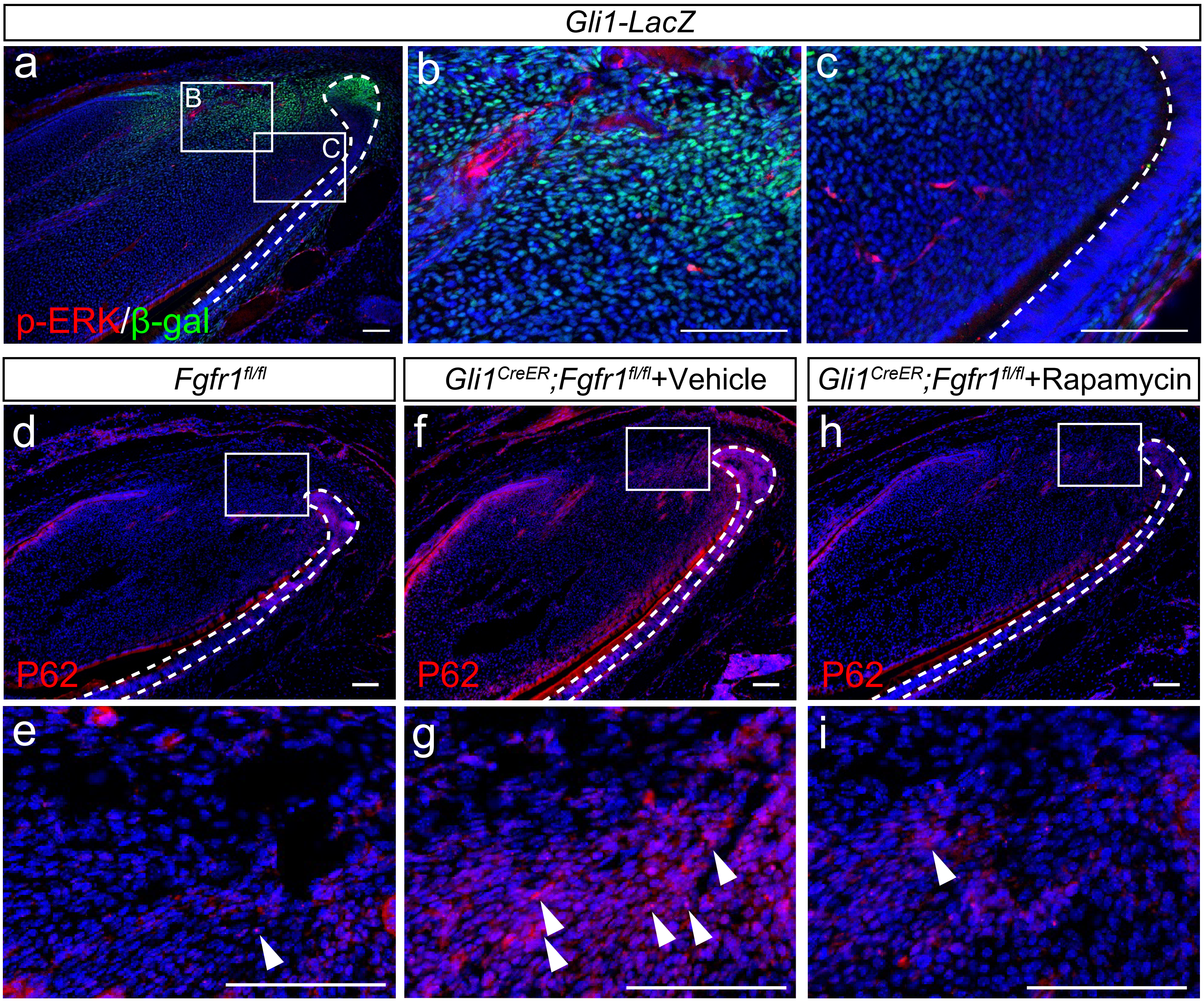
Gli1-LacZ
a
b
c
B
C
p-ERK/β-gal
Fgfr1fl/fl
Gli1CreER;Fgfr1fl/fl+Vehicle
Gli1CreER;Fgfr1fl/fl+Rapamycin
d
f
h
P62
P62
P62
e
g
i

Supplementary Fig. S7: Autophagy is reactivated in *Gli1^{CreER};Fgfr1$^{fl/fl}$* mice by rapamycin injection.

a-c Expression pattern of p-ERK and β-gal in the incisor of *Gli1-LacZ* mice. **d-i** Expression of autophagy substrate P62 in incisors of control, *Gli1^{CreER};Fgfr1$^{fl/fl}$* mice treated with vehicle and *Gli1^{CreER};Fgfr1$^{fl/fl}$* mice treated with rapamycin. White arrowhead points to P62^{+} cells. Scale bars, 100μm.

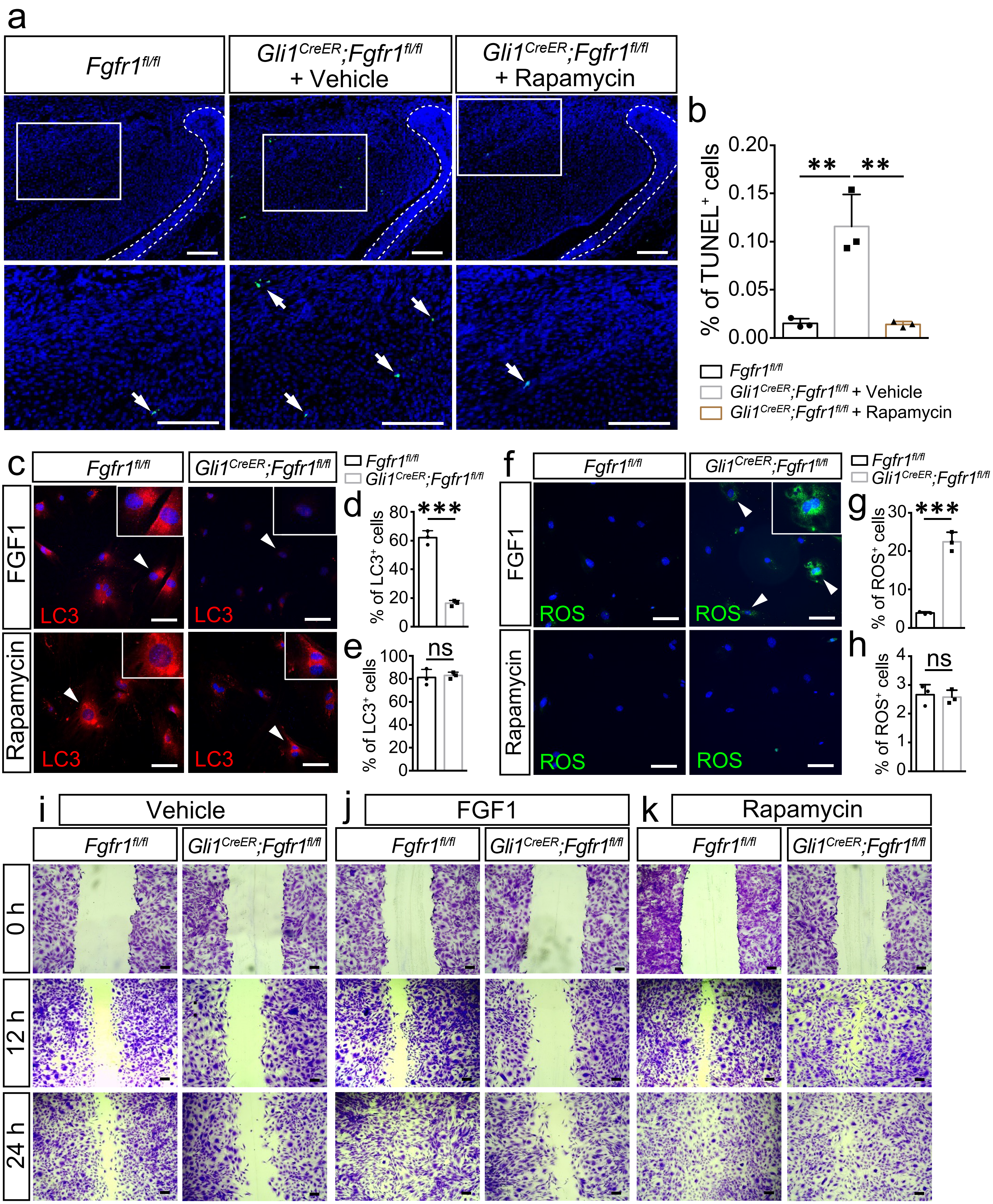
a
Fgfr1 fl/fl
Gli1 CreER;Fgfr1 fl/fl + Vehicle
Gli1 CreER;Fgfr1 fl/fl + Rapamycin
b
% of TUNEL+ cells
0.20
0.15
0.10
0.05
0.00
**
**
Fgfr1 fl/fl
Gli1 CreER;Fgfr1 fl/fl + Vehicle
Gli1 CreER;Fgfr1 fl/fl + Rapamycin
c
FGF1
Rapamycin
LC3
d
% of LC3+ cells
***
e
ns
f
ROS
g
% of ROS+ cells
***
h
ns
i
Vehicle
j
FGF1
k
Rapamycin
0 h
12 h
24 h

Supplementary Fig. 8: Activation of autophagy provides resistance to apoptosis and promotes cell migration.

a $TUNEL^+$ cells in incisors of control, *Gli1^{CreER};Fgfr1$^{fl/fl}$* mice treated with vehicle and *Gli1^{CreER};Fgfr1$^{fl/fl}$* mice treated with rapamycin 3 days after tamoxifen induction. White arrows point to $TUNEL^+$ cells. **b** Quantification of $TUNEL^+$ cells in the mesenchyme. Control versus *Gli1^{CreER};Fgfr1$^{fl/fl}$* + Veh: P=0.0018; *Gli1^{CreER};Fgfr1$^{fl/fl}$* + Veh vs *Gli1^{CreER};Fgfr1$^{fl/fl}$* + Rap: P=0.0017. N=3, each data point represents one animal, with one-way ANOVA analysis. **c** Expression of LC3 in MSCs from control and *Gli1^{CreER};Fgfr1$^{fl/fl}$* mice with FGF1 or rapamycin treatment. White arrowheads point to cells in inset. **d** Quantification of $LC3^+$ cells in control and mutant cells with FGF1 treatment. P=0.0001. **e** Quantification of $LC3^+$ cells in control and mutant cells with Rapamycin treatment. P=0.7155. **f** Expression of ROS in MSCs from control and *Gli1^{CreER};Fgfr1$^{fl/fl}$* mice with FGF1 or rapamycin treatment. White arrowheads point to ROS^+ cells. **g** Quantification of ROS^+ cells in control and mutant cells with FGF1 treatment. P=0.0002. **h** Quantification of ROS^+ cells in control and mutant cells with Rapamycin treatment. P=0.7427. **i-k** Migration of cells from control and *Gli1^{CreER};Fgfr1$^{fl/fl}$* mice treated with vehicle, FGF1 or rapamycin for 0h, 12h and 24h. For **d, e, g** and **h**, n=3, each data point represents one biological replicate, with unpaired Student's t-test performed. All data are expressed as the mean±SD. Source data are provided as a Source Data file. **P<0.01, ****P<0.0001. Each experiment was repeated independently three times. White dotted line shows the cervical loop. Scale bars, 100μm.
