# Supplementary material for: Sensory nerve niche regulates mesenchymal stem cell homeostasis via FGF/mTOR/autophagy axis
Source: Nat Commun. 2023 Jan 20;14:344. doi: 10.1038/s41467-023-35977-4 (PMC9859800; doi:10.1038/s41467-023-35977-4)
Supplement: Supplementary file 3 — Description of Additional Supplementary Files [file 41467_2023_35977_MOESM3_ESM.pdf]

Description of Additional Supplementary Files

Supplementary Movie 1: 3D image of nerves in the incisor with tissue clearing.
