# Supplementary material for: Sensory nerve niche regulates mesenchymal stem cell homeostasis via FGF/mTOR/autophagy axis
Source: Nat Commun. 2023 Jan 20;14:344. doi: 10.1038/s41467-023-35977-4 (PMC9859800; doi:10.1038/s41467-023-35977-4)
Supplement: Supplementary file 5 — Reporting Summary [file 41467_2023_35977_MOESM5_ESM.pdf]

nature portfolio

Corresponding author(s): Yang Chai

Last updated by author(s): Dec 10, 2022

Reporting Summary

Nature Portfolio wishes to improve the reproducibility of the work that we publish. This form provides structure for consistency and transparency in reporting. For further information on Nature Portfolio policies, see our Editorial Policies and the Editorial Policy Checklist.

Statistics

For all statistical analyses, confirm that the following items are present in the figure legend, table legend, main text, or Methods section.

n/a	Confirmed	
☐	☒	The exact sample size (*n*) for each experimental group/condition, given as a discrete number and unit of measurement
☒	☐	A statement on whether measurements were taken from distinct samples or whether the same sample was measured repeatedly
☐	☒	The statistical test(s) used AND whether they are one- or two-sided<br>*Only common tests should be described solely by name; describe more complex techniques in the Methods section.*
☒	☐	A description of all covariates tested
☒	☐	A description of any assumptions or corrections, such as tests of normality and adjustment for multiple comparisons
☒	☐	A full description of the statistical parameters including central tendency (e.g. means) or other basic estimates (e.g. regression coefficient) AND variation (e.g. standard deviation) or associated estimates of uncertainty (e.g. confidence intervals)
☐	☒	For null hypothesis testing, the test statistic (e.g. *F*, *t*, *r*) with confidence intervals, effect sizes, degrees of freedom and *P* value noted<br>*Give P values as exact values whenever suitable.*
☒	☐	For Bayesian analysis, information on the choice of priors and Markov chain Monte Carlo settings
☒	☐	For hierarchical and complex designs, identification of the appropriate level for tests and full reporting of outcomes
☒	☐	Estimates of effect sizes (e.g. Cohen's *d*, Pearson's *r*), indicating how they were calculated

Our web collection on statistics for biologists contains articles on many of the points above.

Software and code

Policy information about availability of computer code

Data collection	single nuclei RNA-sequencing was performed by Novaseq system (Los Angeles, CA), bulk RNA-seq was performed by NextSeq500 system (Los Angeles, CA)
Data analysis	Prism 9 (GraphPad) were used to analyze the data. Partek Flow and IPA were used to analyze bulk RNA-seq data. R Packages Seurat 4.0, CellChat were used to analyze the scRNA-seq data in this study.

For manuscripts utilizing custom algorithms or software that are central to the research but not yet described in published literature, software must be made available to editors and reviewers. We strongly encourage code deposition in a community repository (e.g. GitHub). See the Nature Portfolio guidelines for submitting code & software for further information.

Data

Policy information about availability of data

All manuscripts must include a data availability statement. This statement should provide the following information, where applicable:

- Accession codes, unique identifiers, or web links for publicly available datasets
- A description of any restrictions on data availability
- For clinical datasets or third party data, please ensure that the statement adheres to our policy

Single nuclei RNA-sequencing (snRNA-Seq) and bulk RNA-seq datasets are available through the GEO database under accession code GSE197787; https://www.ncbi.nlm.nih.gov/geo/query/acc.cgi?acc=GSE197787 (the reviewer token is yrapuksapdsznqn)

Field-specific reporting

Please select the one below that is the best fit for your research. If you are not sure, read the appropriate sections before making your selection.

☒ Life sciences ☐ Behavioural & social sciences ☐ Ecological, evolutionary & environmental sciences

For a reference copy of the document with all sections, see nature.com/documents/nr-reporting-summary-flat.pdf

Life sciences study design

All studies must disclose on these points even when the disclosure is negative.

Sample size	sample size was chosen based on previous methods.
Data exclusions	Genes with an expression ratio of <0.1 % and cells with less than 200 were removed.
Replication	All results presented in manuscript were reliably reproduced. Wet lab experiments are representative of 3 independent experiments.
Randomization	Mice from each group were randomly selected for experiments.
Blinding	No blinding were used in this study.

Reporting for specific materials, systems and methods

We require information from authors about some types of materials, experimental systems and methods used in many studies. Here, indicate whether each material, system or method listed is relevant to your study. If you are not sure if a list item applies to your research, read the appropriate section before selecting a response.

Materials & experimental systems

n/a	Involved in the study	
☐	☒	Antibodies
☒	☐	Eukaryotic cell lines
☒	☐	Palaeontology and archaeology
☐	☒	Animals and other organisms
☒	☐	Human research participants
☒	☐	Clinical data
☒	☐	Dual use research of concern

Methods

n/a	Involved in the study	
☒	☐	ChIP-seq
☒	☐	Flow cytometry
☒	☐	MRI-based neuroimaging

Antibodies

Antibodies used	Antibodies used in this study and are listed in Materials and Methods of the manuscript
Validation	Antibodies were used for applications that were validated by the manufacturer.

Animals and other organisms

Policy information about studies involving animals; ARRIVE guidelines recommended for reporting animal research

Laboratory animals	Laboratory animals used in this study and are listed in Materials and Methods of the manuscript
Wild animals	The study did not involve wild animals
Field-collected samples	The study did not involve samples collected from the field
Ethics oversight	All mouse procedures were approved by the Department of Animal Resources and Institutional Animal Care and Use Committee of the University of Southern California (protocol 9320 and 11765).

Note that full information on the approval of the study protocol must also be provided in the manuscript.

March 2021
