# Supplementary figures and images for: Sensory nerve niche regulates mesenchymal stem cell homeostasis via FGF/mTOR/autophagy axis
Source: Nat Commun. 2023 Jan 20;14:344. doi: 10.1038/s41467-023-35977-4 (PMC9859800; doi:10.1038/s41467-023-35977-4)

Fig. 4d

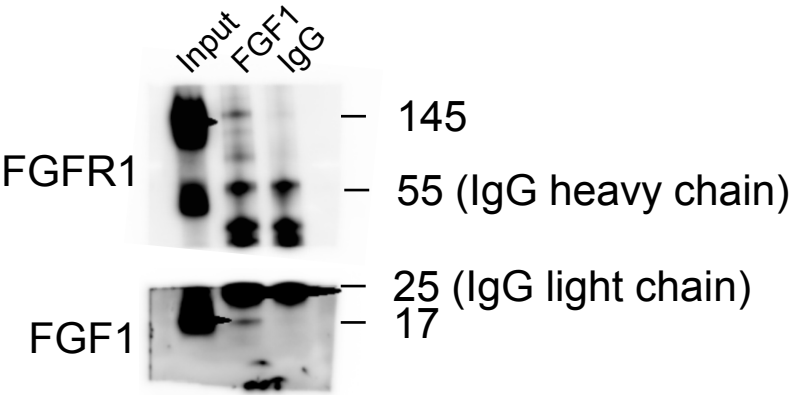

Fig. 6d

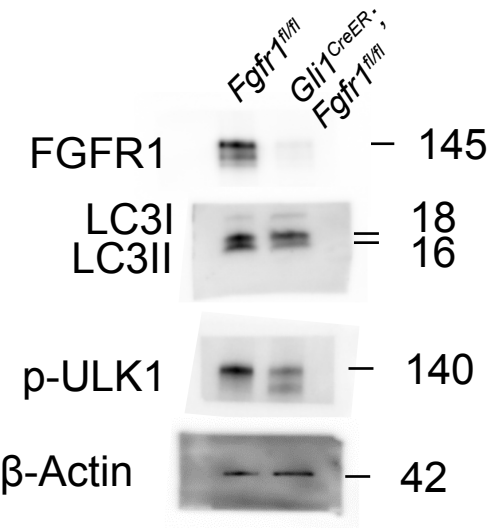

Fig. 6i

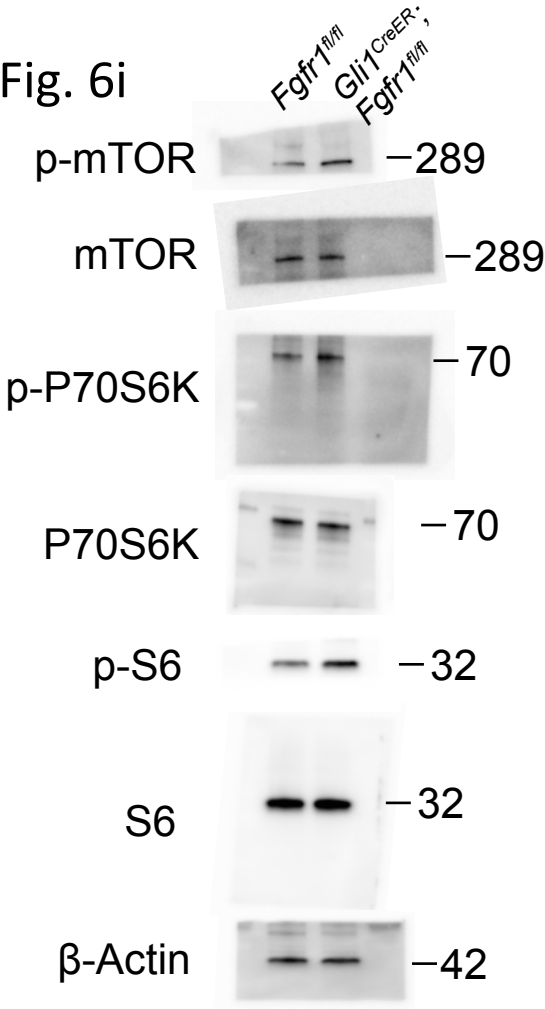

Fig. 6j

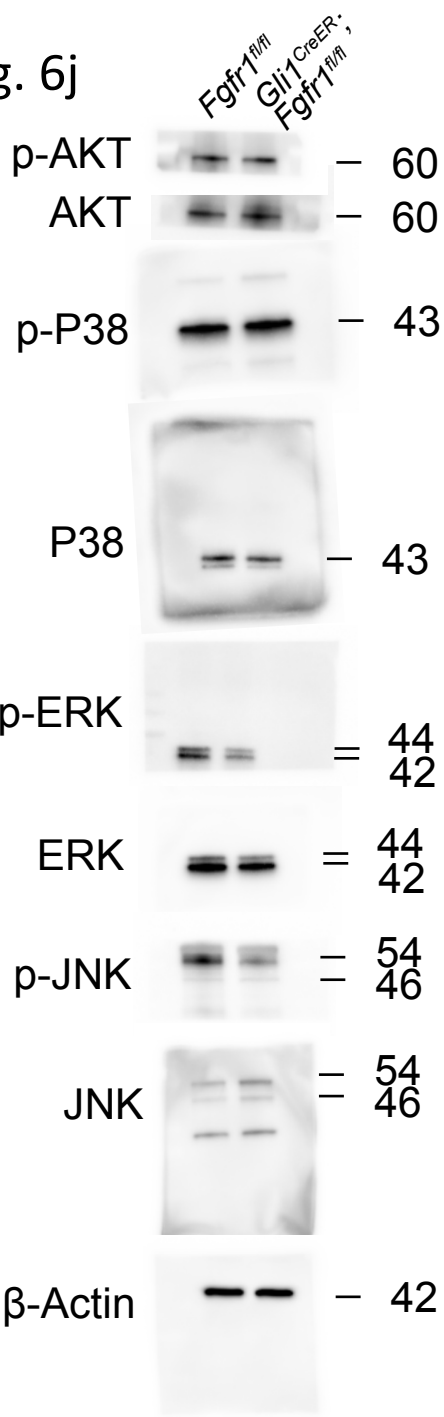

Supplement: Supplementary file 6 — Source Data [file 41467_2023_35977_MOESM6_ESM.zip › uncropped blots.pdf]
